# Reflective optical imaging for scattering medium using chaotic laser

**DOI:** 10.1117/1.JBO.29.4.046008

**Published:** 2024-04-24

**Authors:** Han Lu Feng, Ling Zhen Yang, Jia Li, Zhi Chao Shao, Yi Song Li, Juan Fen Wang, Gang Ti

**Affiliations:** a Taiyuan University of Technology, College of Electronic Information and Optical Engineering, Taiyuan, China; b Taiyuan University of Technology, Ministry of Education, Key Laboratory of Advanced Transducers and Intelligent Control System, Taiyuan, China; c Shanxi Bethune Hospital, Shanxi Academy of Medical Sciences, Department of Medical Record, Taiyuan, China

**Keywords:** optical imaging, reconstruction, correlation, chaotic laser

## Abstract

**Significance:**

Optical imaging is a non-invasive imaging technology that utilizes near-infrared light, allows for the image reconstruction of optical properties like diffuse and absorption coefficients within the tissue. A recent trend is to use signal processing techniques or new light sources and expanding its application.

**Aim:**

We aim to develop the reflective optical imaging using the chaotic correlation technology with chaotic laser and optimize the quality and spatial resolution of reflective optical imaging.

**Approach:**

Scattering medium was measured using reflective configuration in different inhomogeneous regions to evaluate the performance of the imaging system. The accuracy of the recovered optical properties was investigated. The reconstruction errors of absorption coefficients and geometric centers were analyzed, and the feature metrics of the reconstructed images were evaluated.

**Results:**

We showed how chaotic correlation technology can be utilized for information extraction and image reconstruction. This means that a higher signal-to-noise ratio and image reconstruction of inhomogeneous phantoms under different scenarios successfully were achieved.

**Conclusions:**

This work highlights that the peak values of correlation of chaotic exhibit smaller reconstruction error and better reconstruction performance in optical imaging compared with reflective optical imaging with the continuous wave laser.

## 1 Introduction

 Optical imaging is a non-invasive imaging technology that utilizes near-infrared (NIR) light with wavelengths ranging from 600  nm∼1100  nm.^1^^–^^4^ NIR light is highly sensitive to changes in tissue optical parameter,^5^^,^^6^ which allows for the reconstruction of optical properties like diffuse and absorption coefficients within the tissue. Optical imaging provides high spatial resolution and image quality and makes it a valuable tool in various bio-imaging fields.^7^^,^^8^ It has been effectively utilized in applications, such as breast cancer diagnosis, brain function imaging, human joint detection, and thyroid diagnosis.^9^^–^^11^

Optical imaging configurations are divided into the parallel plate, ring and reflective configuration, according to the source and detector placement.^12^^–^^14^ Parallel plate transmission configuration is similar to X-ray mammography. It allows for effective imaging of the breast and detection of abnormalities.^15^^,^^16^ Ring configuration is suitable for mammogram and functional brain imaging. This configuration offers the advantage of capturing images from multiple angles and provides comprehensive information about the detection tissue.^17^ The reflective configuration is commonly used in handheld probes for monitoring cerebral cortex or breast tissue. The light is delivered to the tissue surface by the source, and the reflected light is detected by the adjacent detector.^18^^,^^19^ This configuration enables close-proximity imaging and real-time monitoring of tissue function.^20^ Minghang Li et al. proposed a 3D reconstruction method for highly reflective diffuse object.^21^ Sieno et al. opened the way for optical imaging using SiPM reflective mode source detector arrays.^22^ Mimura obtained the first 3D tissue oxygen saturation map of the human thyroid gland using reflective configuration.^23^

Optical imaging in scattering medium is classified into the continuous wave (CW), frequency domain, and time-resolved imaging according to the different source.^24^^–^^26^ The CW imaging is widely studied and utilized due to its short data acquisition time and cost-effectiveness of detection devices.^27^ However, CW optical imaging can only monitor changes in optical properties through intensity variations and result in the low signal-to-noise ratios (SNR) and the limited structural imaging capabilities.^28^ To achieve the high resolution and accuracy in image reconstruction, researchers have developed various methods and algorithms.^29^ By optimizing the position and orientation of the source detector configuration, the amount of data can be reduced and the quality of the imaging can be improved.^30^^,^^31^ The higher reconstruction quality with a high SNR can be achieved by advancements in signal processing techniques. The accuracy and resolution of the reconstructed images can be significantly improved by increasing the SNR.^32^^,^^33^ In our group, chaotic laser is used to enhance the imaging capabilities of optical imaging. Preliminary investigations have verified the potential of the high-quality images of scattering medium.^34^

Chaotic lasers have the noise-like characteristics in the time series and delta-like auto-correlation functions. The correlation characteristics of chaotic lasers have been widely used in radar applications. Rumbaugh proposed a chaotic lidar transmitter based on an ultra-long cavity fiber laser for underwater ranging and imaging applications.^35^ Ning Jiang proposed a pulsed chaotic multiple input multiple output radar system to achieve centimeter-level resolution for multi-target ranging.^36^ Fan-Yi and Jia-Ming investigated the new chaotic radar system and proposed chaotic radar with high distance resolution.^37^

In this paper, we use the chaotic laser as the light source for reflective configuration optical imaging to study the reconstruction performance of the absorption coefficients of scattering medium. The paper is organized as follows. In Sec. 2, describes the experimental setup, including the production of scattering medium and the reflective optical imaging system with chaotic laser. In Sec. 3, the measurements of the peak values of correlation of chaotic in the proposed system are given and image reconstruction is performed. In Sec. 4, we analyze and discuss the reconstruction results. In Sec. 5, we made the conclusions.

## 2 Experimental Setup

### 2.1 Tissue-Like Phantoms

**Fig. 1:**
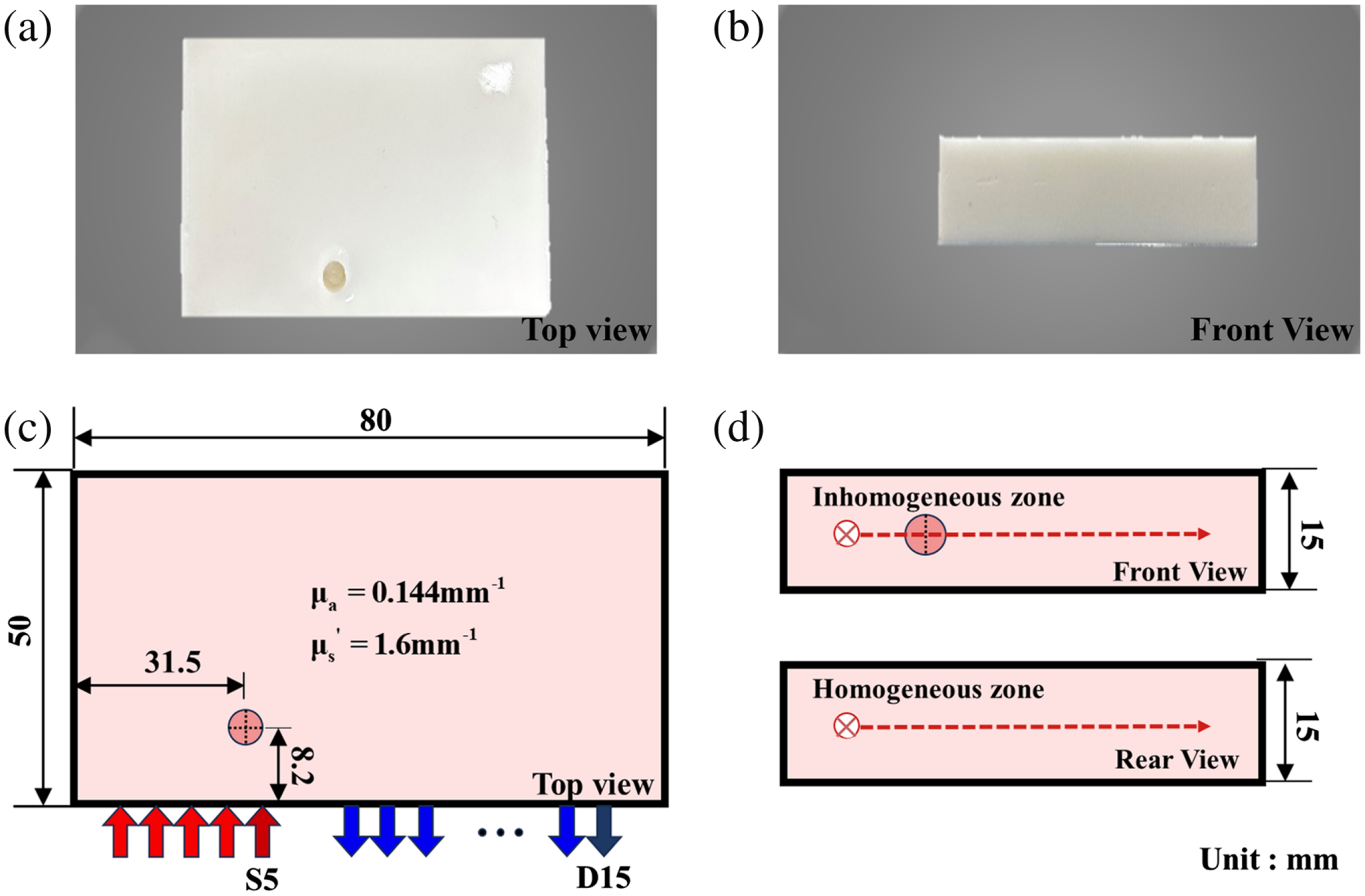
The schematic diagram of tissue-like phantoms (a) top view, (b) front view, (c) reflective configuration, and (d) measurement.

Tissue-like phantoms are used as scattering medium. Agar (Regular Agarose G-10, BIOWEST) powder was used as a coagulant. Water and agar powder were heated and placed in a water bath at a constant temperature of 55°C. Intralipid (100 mL; Intralipid solution 20%) was mixed and stirred with the agar solution and poured into the prepared to produce a tissue matrix of the length of 80.0 mm in, the width of 50.0 mm and the height of 15.0 mm. The top and front views of the tissue-like phantoms are shown in Figs. 1(a) and 1(b). The diffuse and absorption coefficients of the background tissues are $1.60\;{\mathrm{mm}}^{-1}$ and $0.02\;{\mathrm{mm}}^{-1}$ at 1070.22 nm, respectively. A specific concentration of India ink (CM0544, BR, DULY) mixed with 20% Intralipid to simulate the inhomogeneous region is injected into a hole with diameter of 5.0 mm in the tissue-like phantoms. The absorption coefficient of inhomogeneous regions is $0.144\;{\mathrm{mm}}^{-1}$.

The reflective S-D configuration is shown in Fig. 1(c), where the laser source (S1-S5) and detector (D1-D15) are placed on the same side of the tissue phantom, laser source in steps of 1 mm, detector in steps of 2 mm. The laser source irradiated the phantom, and the detector collected the chaotic laser. The time-series of detection signal with different chaotic laser sources were measured at fifteen detection positions. Measurements of the homogeneous and inhomogeneous phantoms are shown in Fig. 1(d).

The cross-correlation function is a statistical representation used to analyze the degree of similarity between the time series of two signals. The cross-correlation function R(τ) between the detection signal and the reference signal can be expressed as

$$R(\tau )=\langle [I(t)-\langle I(t)\rangle ][I^{\prime }(t-\tau )-\langle I^{\prime }(t-\tau )\rangle ]\rangle ,$$ (1)

where I(t) is the detection signal and $I^{\prime }(t)$ is the reference signal. ⟨ ⟩ represents the mean value, t is the time, and τ is the time delay of the two signals. According to the properties of the delta-like cross-correlation of chaotic laser, R(τ) is an ideal delta function. The peak value of the cross-correlation function R(τ) is related to the intensity of the detection signal and the reference signal of the chaotic signals. When the reference signal of the chaotic signals remains unchanged, the peak value of the cross-correlation function R(τ) is related to the changes of the detection signal caused by the scattering and absorption of the tissue phantom. For reflective optical imaging system with chaotic laser, image reconstruction is achieved by the measured peak values of the cross-correlation function R(τ) as boundary measurements at the reflection boundaries.

### 2.2 Experimental Setup of Chaotic Laser

**Fig. 2:**
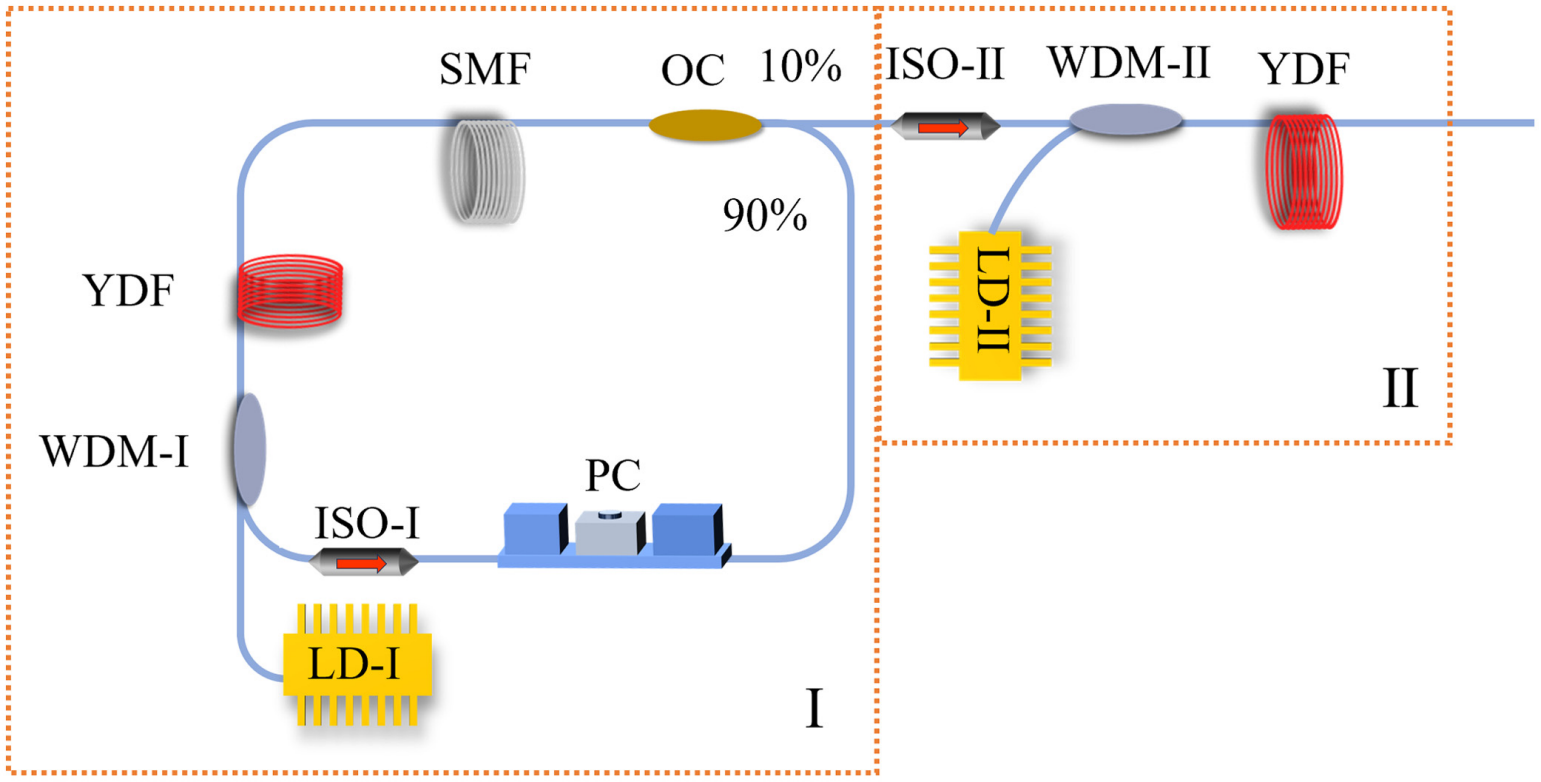
Experimental setup of chaotic laser.

The ytterbium-doped fiber (YDF, SM-YSF-LO-HP, NUFERN) chaotic laser system consists of a chaotic laser source (I) and an amplifier (II) as shown in Fig. 2. The 976 nm optical signal generated by a laser diode (LD-I, LC96Z6OO-74) passes through a fiber ring cavity, which is composed of a wavelength division multiplexer (WDM-I, WDM-1×2-980/1064-0-A55), YDF, single-mode fiber (SMF, 160-XP, NUFERN), polarization-independent isolator (ISO-I, I-1064-00-S-C-10-L-N), polarization controller (PC, FPC-100, Thorlabs), and 90:10 optical coupler (OC, WBC-1×2-1060-10/90). The chaotic laser is generated based on the nonlinear effect by adjusting the pump current and the polarization in the cavity. The chaotic laser is then coupled into the YDF through an ISO-II (I-1064-00-S-C-10-L-N) and WDM-II (WDM-1×2-980/1064-0-A55). LD-II (LC96Z6OO-74) at 976 nm is used for amplification of the chaotic laser source.

### 2.3 Reflective Optical Imaging System With Chaotic Laser

**Fig. 3:**
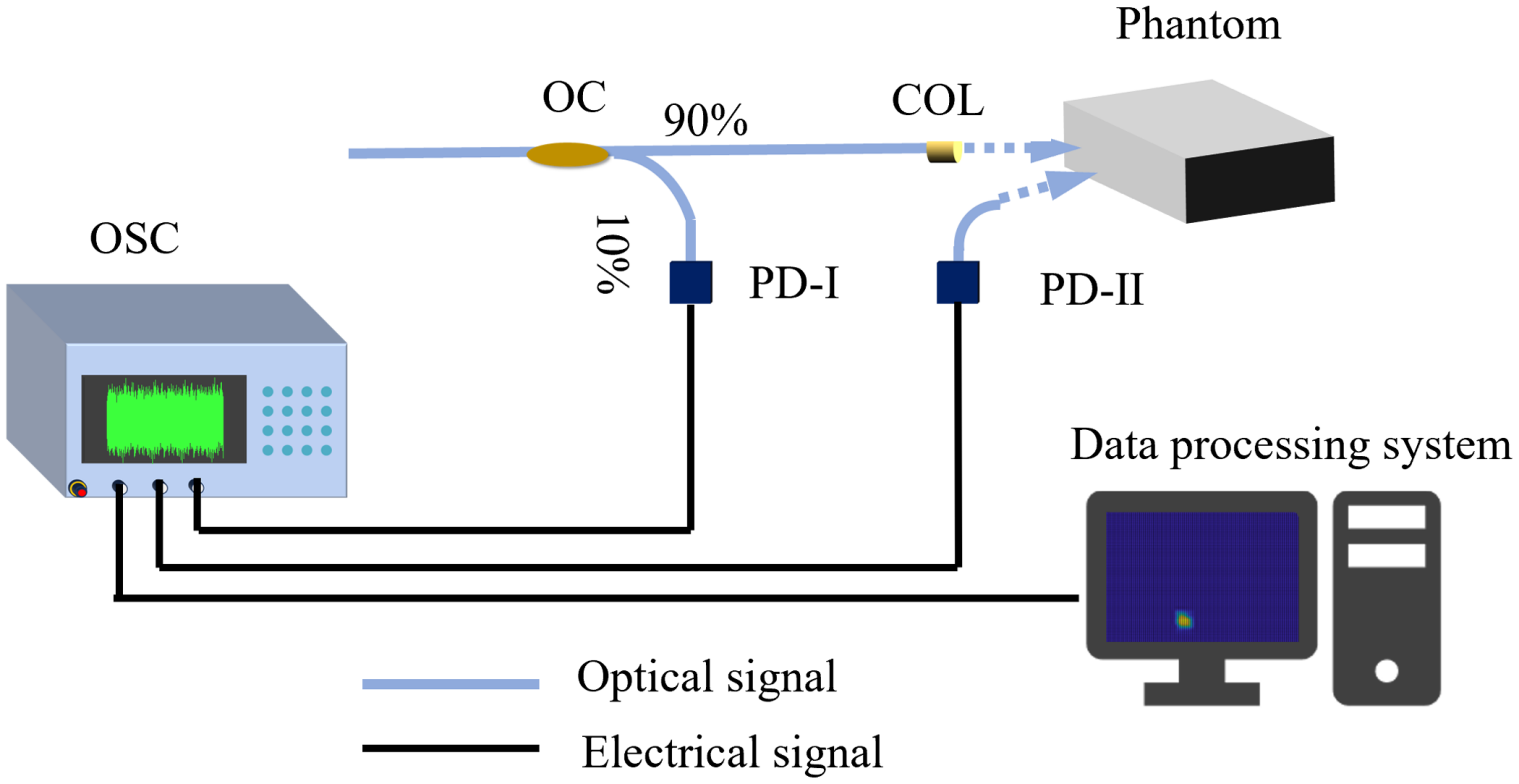
Reflective optical imaging system with chaotic laser.

The reflective optical imaging system with chaotic laser is shown in Fig. 3. The chaotic laser was split into the detection and reference signal with a 90:10 OC (WBC-1×2-1060-10/90). The reference optical signal has a power of 13 mW and is detected by photoelectric detector PD-I (DET01CFC, THORLABS). The collimated chaotic laser source with the power of 115 mW illuminates the tissue phantoms. The detection signal is detected by the photoelectric detector (PD-II, PDA05CF2, THORLABS) mounted on a motorized precision displacement stage. The reference signal I’(t) is measured by PD-I and the detection signal I(t) is measured by PD-II. The electrical signals through PD-I and PD-II are recorded with the oscilloscope (OSC, MSO64, TEKTRONIX) at the same time. The cross-correlation between the electric signals of the output of PD-I and PD-II is carried out by the data processing system.

## 3 Experimental Result

### 3.1 Characteristics of Chaotic Laser

**Fig. 4:**
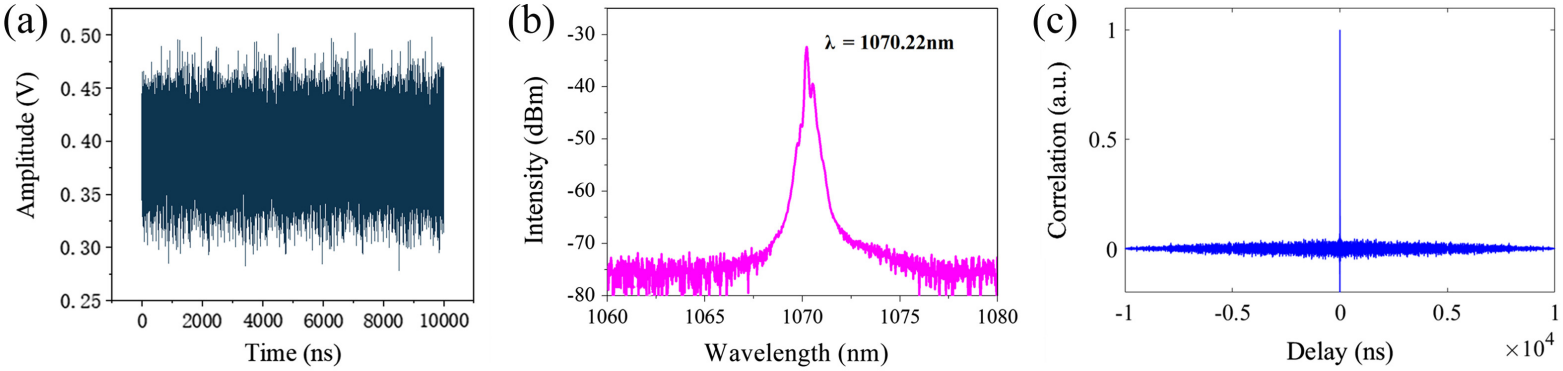
The chaotic laser of (a) time series, (b) optical spectrum, and (c) correlation curve.

The time series of the chaotic laser is shown in Fig. 4(a), which the intensity varies randomly with time. The optical spectrum in Fig. 4(b) shows that the central wavelength of the chaotic laser is 1070.22 nm. The delta-like characteristics of the ytterbium-doped chaotic laser are obtained by the auto-correlation algorithm and are shown in Fig. 4(c). These results highlight the properties exhibited by the chaotic laser and provide the further insights into the nature of the chaotic laser.

**Fig. 5:**
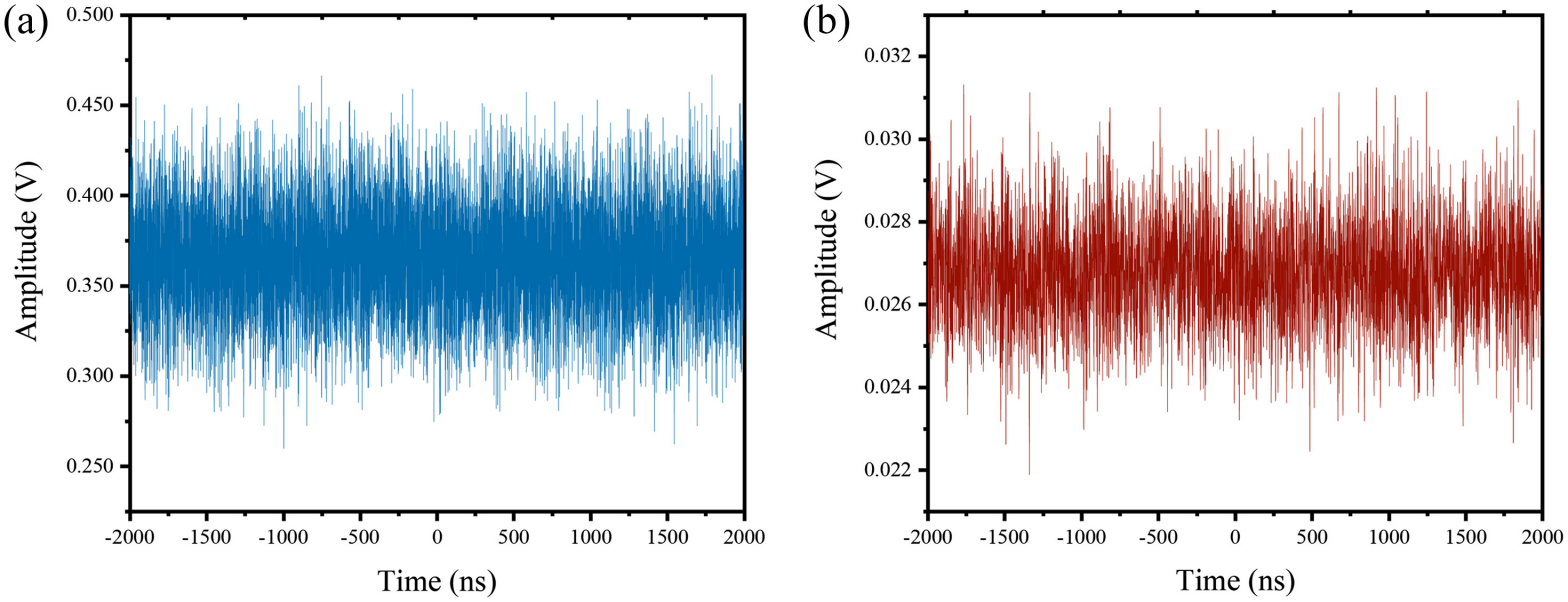
The time series of (a) detection signal and (b) reference signal.

The detection and reference signal measured by the PD-II and PD-I are shown in Fig. 5. Figure 5(a) shows the time series of the detection signal and the time series is random. Figure 5(b) shows the time series of the reference signal and time series is also random.

### 3.2 Correlation Characteristics of Chaotic Laser in Inhomogeneous Regions

The experiments by scattering tissue inhomogeneous with mixtures of India ink and Intralipid in a certain concentration. The single inhomogeneous phantom and multiple-inhomogeneous phantom were used to analyze the correlation characteristics of chaotic laser after scattering. The absorption and scattering coefficients of the inhomogeneous phantom are $1.600\;{\mathrm{mm}}^{-1}$ and $0.144\;{\mathrm{mm}}^{-1}$. Respectively, the depth of the inhomogeneous hole of single inhomogeneous phantom is 8.2 mm, the coordinates of center of the inhomogeneous hole were (−8.5,8.2). In the multi-inhomogeneous phantoms, the depth of the inhomogeneous hole is 14.0 mm, the coordinates of the two inhomogeneous holes were (−10.5,14.0) and (−3.5,14.0). The geometric centers of these regions were 7 mm apart, with a minimum distance of 2 mm.

**Fig. 6:**
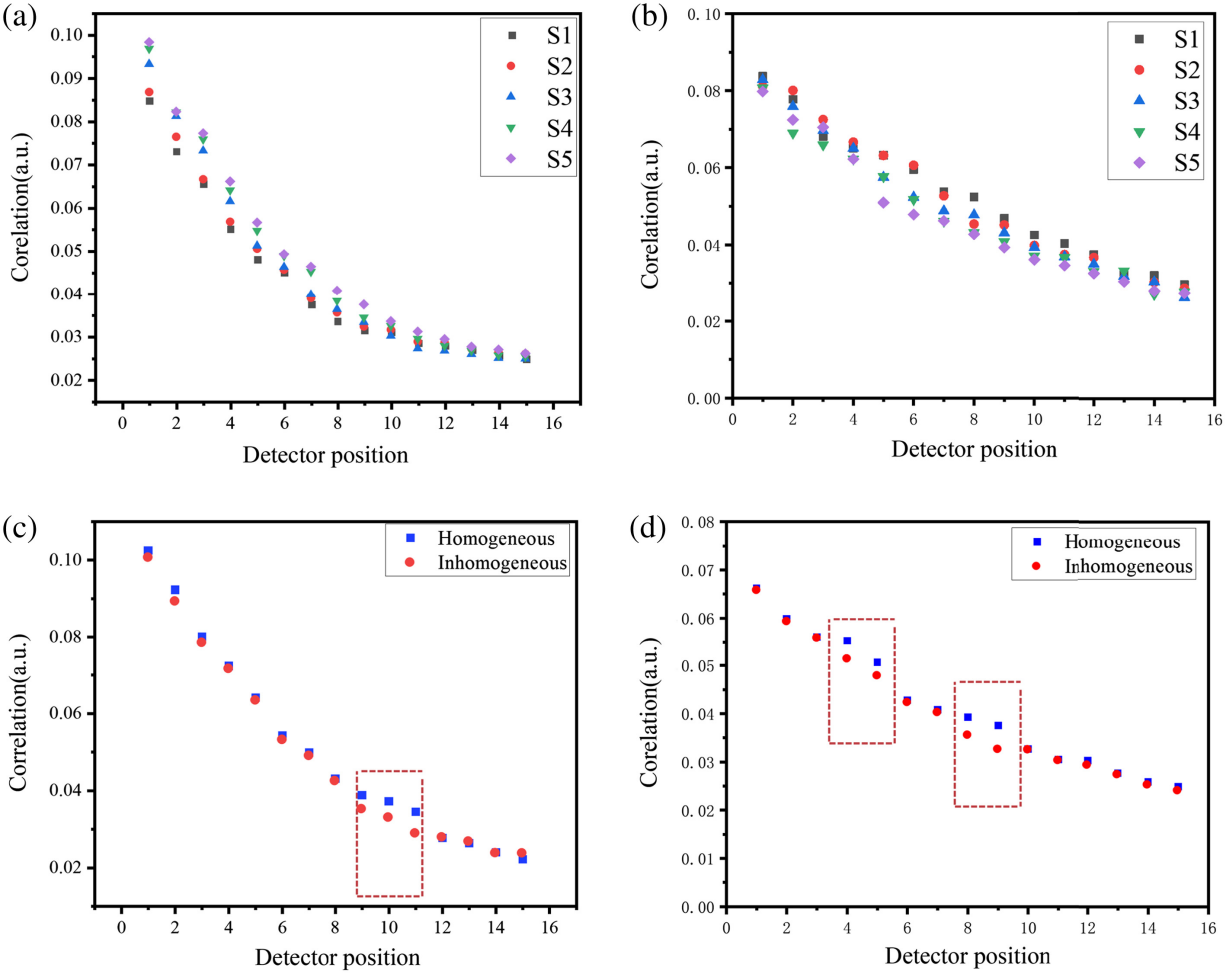
The peak values of correlation of chaotic laser with detection position (a) single inhomogeneous and (b) multiple inhomogeneous. The comparison curve (c) single inhomogeneous and (d) multiple inhomogeneous.

The detection signal of chaotic laser I(t) for the single and multiple-inhomogeneous phantom after scattering was measured experimentally and cross-correlated with the reference laser $I^{\prime }(t)$. The peak values of cross-correlation of chaotic laser R(τ) of inhomogeneous phantom in the case of five laser sources are measured in Figs. 6(a) and 6(b), and it indicated that the peak values of correlation of chaotic laser decreases with increasing S-D distance. Figures 6(c) and 6(d) show a comparison of the peak values of chaotic correlation in homogeneous and inhomogeneous regions. Due to the high absorption characteristics of the inhomogeneous region for NIR light, the peak values of correlation of chaotic are clearly different at detector positions corresponding to the high absorption regions. The peak values of chaotic correlation demonstrate the corresponding reflectance measurements and illustrate the valid information of the inhomogeneous regions.

**Fig. 7:**
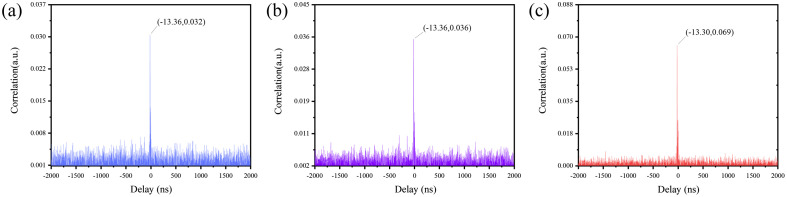
The correlation curve in S1-D10 (a) inhomogeneous and (b) homogeneous; S1-D4 (c) inhomogeneous.

Figures 7(a) and 7(b) show the correlation curve of S1-D10 positions in the case of a single inhomogeneous region. The coordinate of the peak in Fig. 7(a) is (−13.36,0.032) and it indicates that the time delay τ between the detection signal and reference signal caused by the distance difference between the two signals is −13.36  ns and 0.032 is the peak value of cross-correlation. The coordinate of the peak in Fig. 7(b) is (−13.36,0.036) and it indicates that the absorption and scattering of the phantom are higher in the inhomogeneous region than in the homogeneous region. Figure 7(c) demonstrates the correlation curve of S1-D4 positions in the case of a single inhomogeneous region and the coordinate of the peak is (−13.30,0.069). The peak value of cross-correlation of the homogeneous region is the same with the Fig. 7(c), and it is because the distribution of the absorption and scattering coefficients in homogeneous region differs only in the hole of inhomogeneous region in the tissue-like phantoms.

### 3.3 Image Reconstruction Based on the Peak Values of the Correlation

**Fig. 8:**
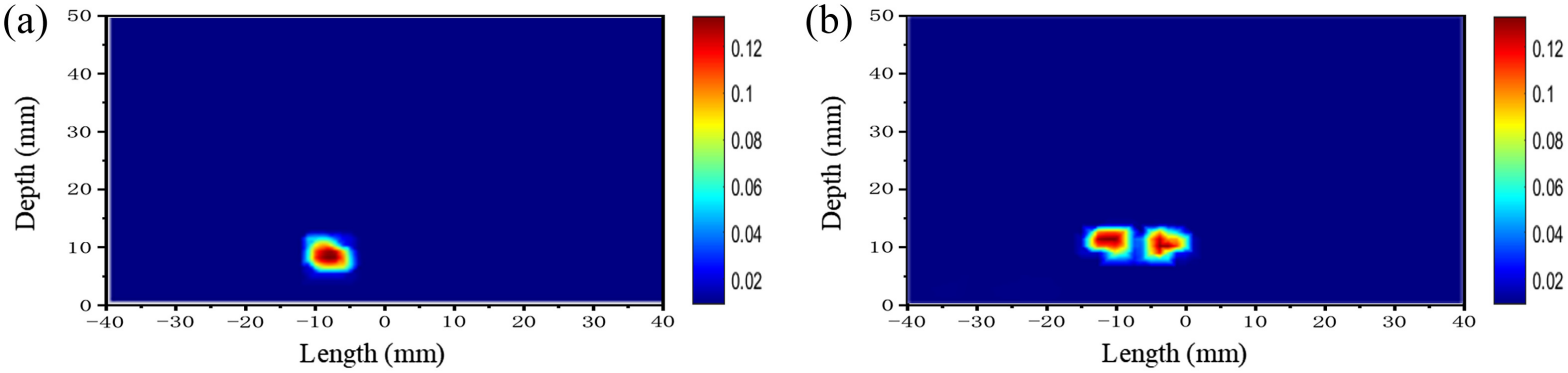
The reconstructed image using the peak values of correlation for (a) single inhomogeneous phantom and (b) multiple inhomogeneous phantoms.

Image reconstruction is defined as the inverse problem of the photon propagation model, which involves the internal spatial distribution of the objective function using boundary measurements of tissue-like phantoms. Diffusion equation is used to describe the transmission of chaotic laser within tissue-like phantoms. We used the finite element method framework for the forward and reverse problems of chaotic laser.^38^ The peak values of correlation of chaotic laser are used as input to the inverse problem of image reconstruction and the recovery of the absorption coefficients in the phantoms. The reconstruction results for single and multiple-inhomogeneous phantom are shown in Fig. 8.

## 4 Discussion

**Fig. 9:**
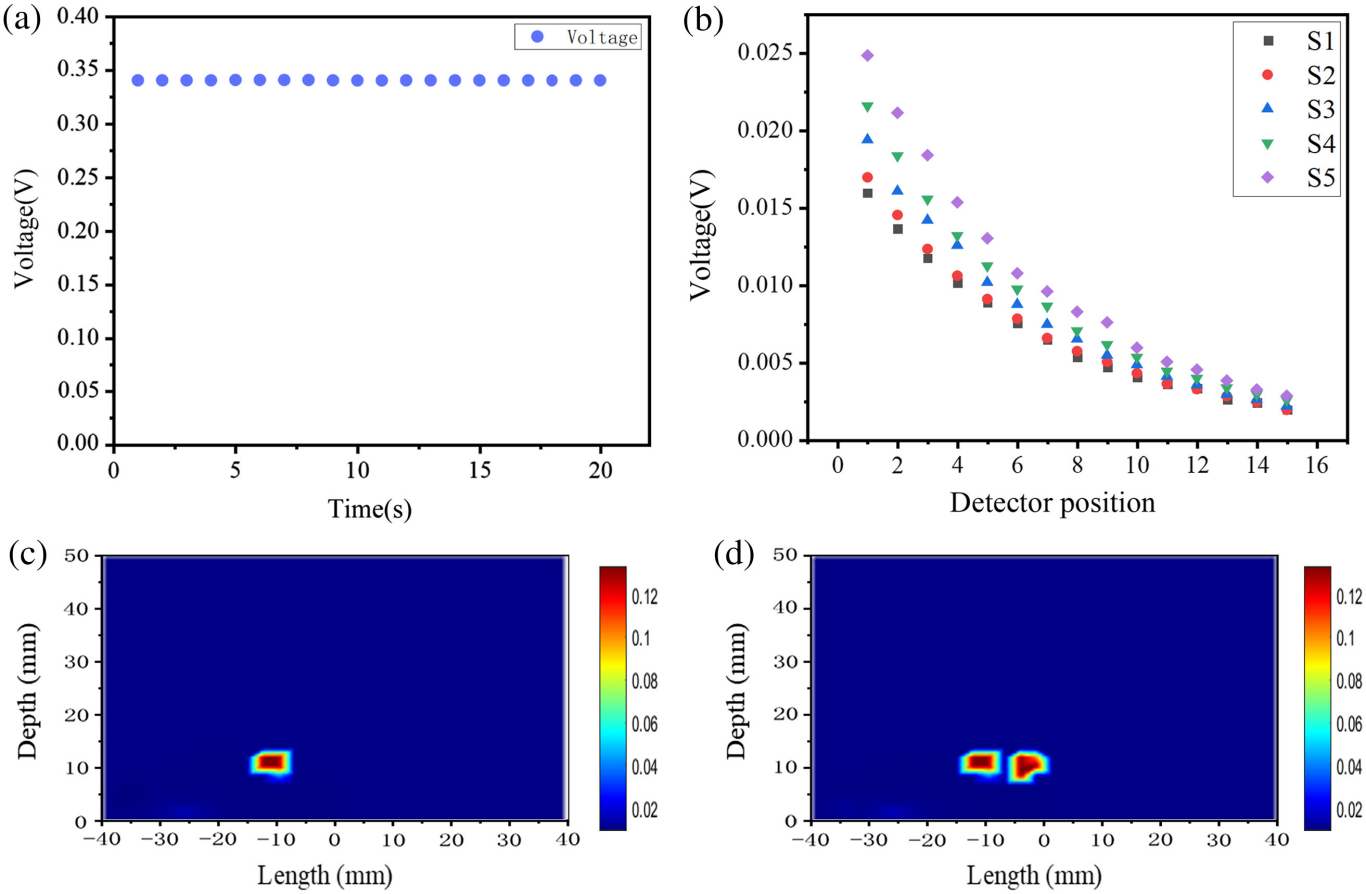
(a) The voltage in time, (b) the output light intensity with detection position, (c) single inhomogeneous phantom, and (d) multiple-inhomogeneous phantom.

To compare with the CW optical imaging, we carried out the experimental analysis of the optical imaging system based on CW laser. The CW laser was obtained by adjusting the polarization control in the ring fiber laser. Figure 9(a) demonstrates that the voltage of output light intensity remains stable with time. Figure 9(b) demonstrates the voltage of the output light intensity with detection position in the measurement with CW laser, and the voltage of output light intensity decreases exponentially with increasing distance between the light source and the detector. Figures 9(c) and 9(d) demonstrates the corresponding image reconstruction results. The presented plots illustrate the characterization in the case of a single inhomogeneous phantom and demonstrate that it is possible to obtain partial information about the inhomogeneous regions.

**Fig. 10:**
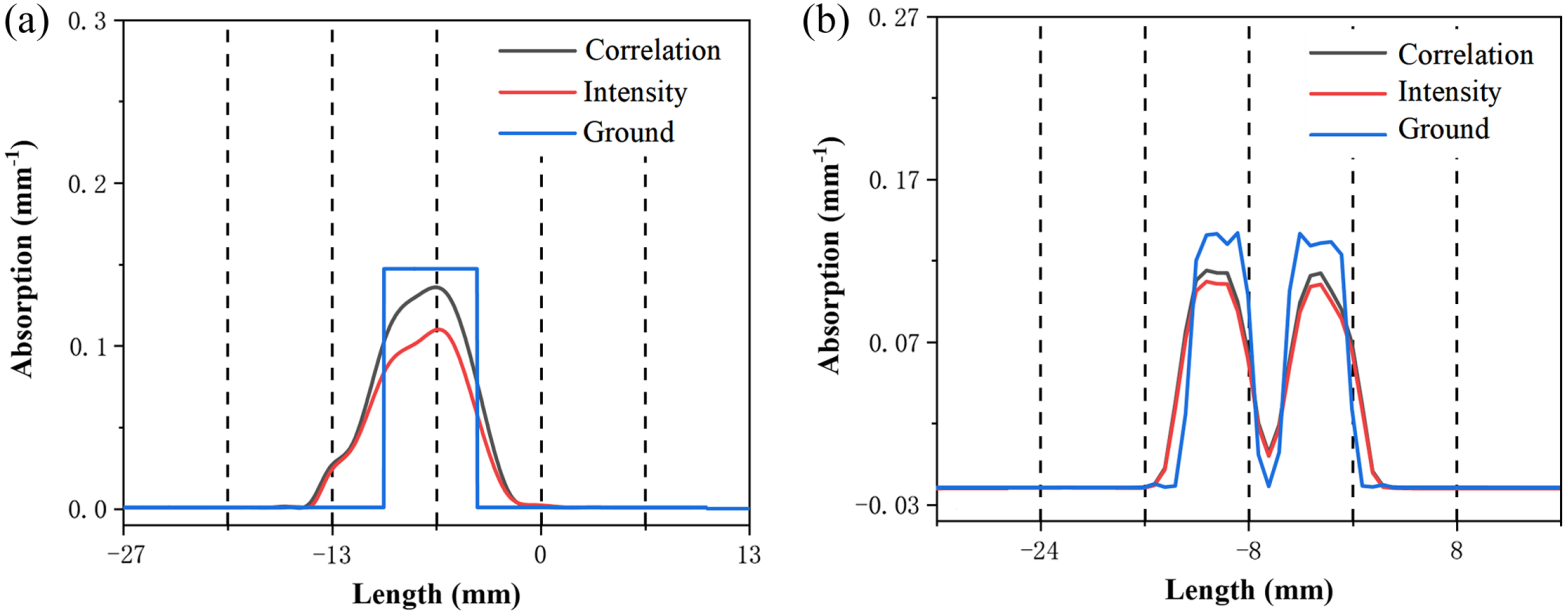
The distribution of absorption coefficient (a) at depth of 10 mm for single inhomogeneous and (b) at depth of 12 mm for multiple inhomogeneous.

Figure 10(a) illustrates the distribution of absorption coefficients extracted at depth of 10 mm in the reconstructed image of a single inhomogeneous phantom. The results of the reconstruction indicate that the maximum absorption coefficient using the peak values of correlation of chaotic laser is $0.121\;{\mathrm{mm}}^{-1}$, the maximum absorption coefficient of image reconstruction with CW is $0.105\;{\mathrm{mm}}^{-1}$. Figure 10(b) shows the absorption coefficients extracted at 12 mm depth of the reconstructed image of multiple inhomogeneous regions in the variation curves. The maximum absorption coefficients of the two inhomogeneous regions are $0.115\;{\mathrm{mm}}^{-1}$ and $0.113\;{\mathrm{mm}}^{-1}$ based on the peak values of correlation of chaotic laser for image reconstruction. The absorption coefficients of the same regions with the intensity reconstruction are $0.101\;{\mathrm{mm}}^{1}$ and $0.105\;{\mathrm{mm}}^{-1}$. The true value of the absorption coefficient is $0.144\;{\mathrm{mm}}^{-1}$. This difference suggests that peak values of correlation of chaotic laser in the reconstruction process can achieve the more accurate estimation of the absorption coefficient.

The analysis is evaluated from reconstruction accuracy and image quality. The reconstruction accuracy is analyzed by the absorption coefficient error and the geometric center error. The distances between these coordinates and the true coordinates were calculated based on the center-of-mass coordinates of the different targets in the optical imaging reconstruction results. The geometric center error δd is defined as

$$\delta d={\left\{{\left(x-x_{0}\right)}^{2}+{\left(y-y_{0}\right)}^{2}\right\}}^{1/2}/d,$$ (2)

where d is the true diameter of the ground. The x and y are the coordinates at the absorption coefficient reconstruction maximum, $x_{0}$ and $y_{0}$ are the true center position coordinates of the ground. For a single inhomogeneous region, δdc is 33.8% and δdi is 34.7%, and c and i represent the correlation and intensity respectively. For multiple inhomogeneous regions, δdc is 52.1% and δdi is 92.3%. The coefficient error δμ is defined as

$$\delta \mu =(\mu -\mu_{a})/\mu ,$$ (3)

where μ and $\mu_{a}$ are the ground truth and reconstructed absorption coefficients. For single inhomogeneous region, $\delta \mu_{c}$ is 12.5% and $\delta \mu_{i}$ is 25.6%. For multiple inhomogeneous regions, $\delta \mu_{c}$ is 20.8% and $\delta \mu_{i}$ is 28.4%. The correlation data of multiple inhomogeneous regions are averaged, and the corresponding analysis results are given in Table 1.

**Table 1:** Results of reconstruction.

	Correlation		Intensity	
	δd	δμ	δd	δμ
Single inhomogeneous	33.8%	12.5%	34.7%	25.6%
Multiple inhomogeneous	52.1%	20.8%	92.3%	28.4%

Due to the combination of chaotic signal and cross-correlation algorithm, the anti-interference capability of the detection signal is effectively improved, which makes the system to have the better localization accuracy and absorption coefficient reconstruction for inhomogeneous regions. Compared with CW laser, the chaotic correlation technology can result in the significant decrease in both geometric center error and absorption coefficient error. For a single inhomogeneous region, the geometric center error and absorption coefficient error are decreased by 1.02 times and 2.04 times, respectively. For multiple inhomogeneous regions, the geometric center error and absorption coefficient error are decreased by 1.77 times and 1.36 times, respectively.

Kullback–Leibler (KL) scatter is employed to measure the similarity between the reconstructed image and the reference image, where smaller values indicate better similarity [31]. Furthermore, the mean square error (MSE) and peak SNR (PSNR) are utilized to evaluate the distortion of the reconstructed image. A smaller MSE and a larger PSNR indicate higher image quality. The effective resolution (ERES) is defined as twice the distance between the center of the actual inhomogeneous region and any node with a value greater than or equal to 50% of the maximum value in the reconstructed image. The performance evaluation results of reconstructed image are given in Table 2.

**Table 2:** Performance evaluation of reconstructed image.

Metrics	Single inhomogeneous regions		Multiple-inhomogeneous regions	
	Correlation	Intensity	Correlation	Intensity
ERES	3.935	4.060	4.785	6.107
MSE	$3.440\times 10^{-6}$	$4.928\times 10^{-5}$	$6.070\times 10^{-5}$	$6.335\times 10^{-5}$
PSNR	31.316	25.530	27.088	21.083
KL	0.039	0.046	0.958	0.974

The ERES of the reconstructed images using peak values of correlation of chaotic laser in both the single and multiple inhomogeneous regions is lower compared to the results with the CW laser and it is indicated the higher reconstruction resolution with the chaotic laser. The ERES in the reconstructed images is also lower than the values reported in the Ref. 39. In terms of MSE, the reconstructed images using the peak values of correlation of chaotic laser show an order of magnitude lower error compared to the CW laser under a single inhomogeneous region, which better represents the real situation. The difference becomes smaller under multiple inhomogeneous regions due to the increased complexity of the phantom. The MSE of the reconstructed images are lower than those reported in the Ref. 40. For the evaluation of PSNR, the peak values of correlation of chaotic laser have significantly higher values compared to the results with CW laser. The KL image similarity assessments demonstrate a high similarity of the reconstructed images using the peak values of chaotic correlation to the actual situation.

## 5 Conclusion

We propose and demonstrate a reflective scattered chaotic laser system for optical imaging. Experimental results validate that the variation of the peak values of correlation coefficient of the chaotic laser adheres to the theoretical model. We successful reconstructions of the absorption coefficient distribution within tissue-like phantoms in both single and multiple inhomogeneous regions. Furthermore, the evaluation of image quality indicates that the unique characteristics of the chaotic laser can be effectively utilized to provide enhanced spatial resolution enhancement and more precise reconstructed images. This novel approach offers a promising detection laser source for non-invasive medical imaging and holds potential applications in various areas such as skin, brain, and joint-related diagnoses.

## Data Availability

Data underlying the results presented in this paper are not publicly available at this time but may be obtained from the authors upon reasonable request.
